# Cheating does not explain selective differences at high and low relatedness in a social amoeba

**DOI:** 10.1186/1471-2148-10-76

**Published:** 2010-03-12

**Authors:** Gerda Saxer, Debra A Brock, David C Queller, Joan E Strassmann

**Affiliations:** 1 Department of Ecology and Evolutionary Biology MS 170, Rice University, 6100 Main Street, Houston, TX 77005, USA

## Abstract

**Background:**

Altruism can be favored by high relatedness among interactants. We tested the effect of relatedness in experimental populations of the social amoeba *Dictyostelium discoideum*, where altruism occurs in a starvation-induced social stage when some amoebae die to form a stalk that lifts the fertile spores above the soil facilitating dispersal. The single cells that aggregate during the social stage can be genetically diverse, which can lead to conflict over spore and stalk allocation. We mixed eight genetically distinct wild isolates and maintained twelve replicated populations at a high and a low relatedness treatment. After one and ten social generations we assessed the strain composition of the populations. We expected that some strains would be out-competed in both treatments. In addition, we expected that low relatedness might allow the persistence of social cheaters as it provides opportunity to exploit other strains.

**Results:**

We found that at high relatedness a single clone prevailed in all twelve populations. At low relatedness three clones predominated in all twelve populations. Interestingly, exploitation of some clones by others in the social stage did not explain the results. When we mixed each winner against the pool of five losers, the winner did not prevail in the spores because all contributed fairly to the stalk and spores. Furthermore, the dominant clone at high-relatedness was not cheated by the other two that persisted at low relatedness. A combination of high spore production and short unicellular stage most successfully explained the three successful clones at low relatedness, but not why one of them fared better at high relatedness. Differences in density did not account for the results, as the clones did not differ in vegetative growth rates nor did they change the growth rates over relevant densities.

**Conclusions:**

These results suggest that social competition and something beyond solitary growth differences occurs during the vegetative stage when amoebae eat bacteria and divide by binary fission. The high degree of repeatability of our results indicates that these effects are strong and points to the importance of new approaches to studying interactions in *D. discoideum*.

## Background

The evolution of altruism is difficult to explain because altruistic acts benefit other individuals while reducing the fitness of the actor. According to Hamilton's rule, cooperative and altruistic interactions are favored by genetic similarity (with sufficient benefits relative to costs) [1]. This is consistent with high levels of relatedness observed in cooperative organisms, such as social insects [2,3] and spores in a fruiting body of the social amoeba *Dictyostelium discoideum *[4,5]. Hamilton proposed that genetic similarity among interactors can be increased if individuals can recognize genetically similar individuals or, more passively, if limited dispersal increases the likelihood that neighbors are genetically similar. This suggests that in spatially structured populations where interactions are often limited to neighbors, interactions are also more likely to take place among genetically similar individuals. Theoretical models suggest that spatial structure can select for the evolution of cooperation but can also increase competition among relatives [6-10]. However, if there is a dispersal stage followed by a high relatedness stage, as is likely to be the case in *D. discoideum*, competition is less likely to counter cooperation. The impact of spatial structure and relatedness on the evolution of social interactions can be studied in microbial experimental systems [11-14].

**Figure 1:**
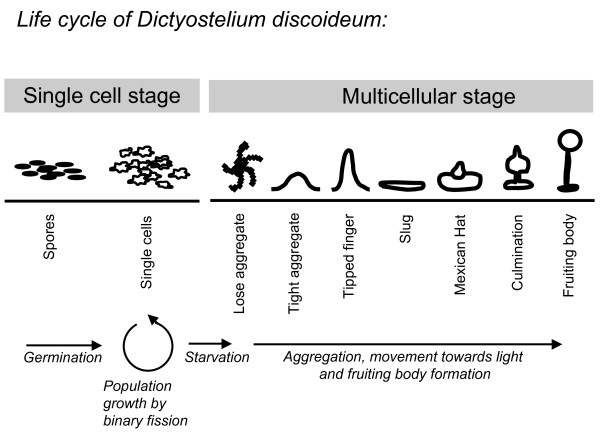
**Life cycle of *Dictyostelium discoideum***. During the unicellular stage of the life cycle, spores germinate into single cells that eat microorganisms and divide by binary fission. Upon starvation, the multicellular stage of the life cycle is initiated. Single cells aggregate and undergo complex development to form a fruiting body structure, which consists of about 20% dead stalk cells and 80% hardy spores.

The social amoeba *Dictyostelium discoideum *is particularly well suited to test the effect of spatial structure and relatedness on the evolution of cooperation, because it has a complex asexual life cycle composed of a unicellular stage and a multicellular stage with clear altruism (Figure 1) [15-17]. During the unicellular, vegetative stage, spores germinate into single-celled amoebae that grow and divide by binary fission. These amoebae live in soil and feed on microorganisms. Upon starvation, the multicellular stage of the life cycle begins. Single cells aggregate and form a motile slug that eventually develops into a fruiting body that consists of a stalk made of dead cells and a sorus containing the fertile spores. During fruiting body formation, about 20% of the cells die to form the stalk. In contrast to other multicellular organisms, the multicellular stage of *D. discoideum *does not go through a single cell bottleneck, but rather is the result of aggregation by thousands of cells. These aggregating amoebae can be genetically distinct from their co-aggregants, which can lead to conflict over spore and stalk allocation [16-18] such that one strain produces proportionately more spores and does not contribute its fair share of dead cells to the stalk. Accordingly, we define a cheater as a strain that increases in frequency during the multicellular stage when aggregating with cells of a genetically distinct strain.

Despite the potential for conflict among aggregating cells, there is a clear benefit gained from the social interaction during the multicellular stage, because *D. discoideum *cells are unable to form spores without the formation of a fruiting body [19]. The development of spores is advantageous, because only spores are able to survive harsh, nutrient deprived and desiccating environments. The stalk of the fruiting body raises the sorus above the ground and increases the likelihood that spores will be dispersed to a nutrient rich patch either through falling a little farther from the stalk or through passive dispersal by animals.

The two stages of the life cycle are dominated by different interactions and selection pressures. During the feeding stage, access to food and protection from predators are likely to be important. Since *D. discoideum *feeds by engulfing bacteria in an essentially solitary process and is not known to mount any group defenses, the impact of social interactions is not expected to be as great as during the social stage when cells cease to grow and 20% of amoebae sacrifice their lives for the benefit of others. Exactly how they compete with each other for food or other resources during the solitary growth stage is not known. Since growth and replication only occur during the solitary stage and cells in nature are likely go through several to hundreds of vegetative generations between social generations, differences during solitary growth may result in strong selection.

To test the effects of relatedness on the evolution of altruism, we created genetically diverse populations by mixing equal numbers of spores of eight genetically distinct wild isolates. These populations were maintained for ten rounds of their life cycle under conditions that promoted social interactions at high or low relatedness. Since the amoebae undergo one multicellular social stage per cycle by definition, we will henceforth refer to one completion of the asexual life cycle as one social generation. We manipulated spatial structure and hence relatedness of aggregating cells by plating the populations at the beginning of each social generation at either high or low density. At high plating density, single cells encounter more non-relatives than at low plating density, which is reflected in lower relatedness among spores in single fruiting bodies at high density.

We manipulated relatedness by manipulating density, so we should consider what effects density alone might have, though nothing specific is documented in the literature on *D. discoideum *densities. In our experimental conditions, the low-density populations will go through a few more vegetative generations per cycle than the high-density populations, giving greater selective opportunity to any clone that grows faster. It is possible that the clones' relative growth rates change with density. To be able to account for possible effects of different plating densities, we measured vegetative growth rates and germination rates of each strain individually. In addition, the low plating densities of spores of the high relatedness populations could be more susceptible to drift. To be able to distinguish the effects of drift and selection on these populations, we performed the experiment with twelve replicated populations at both high and low relatedness.

In most organisms, competition for limited resources and social interactions are highly intertwined, which makes it difficult to disentangle the effects of social traits from other life history traits [20]. Though selection can act on different traits in our experimental system, the temporal separation of the unicellular stage and the social, multicellular stage allows us to test the effects of social traits and other life history traits separately. In addition, by forcing a global dispersal stage between social generations we can avoid complications that arise with limited dispersal, which can increase both relatedness and local competition [6-10].

To assess the success of the different strains in our selection experiment, we measured the frequencies of each strain in the population and estimated genetic diversity by calculating Shannon's Index of diversity (H'), which has its highest value when all strains are present in equal frequencies and declines with the loss of strains or unequal frequencies of the strains. Over the course of ten social generations, we expected that genetic diversity within populations would decline at both high and low relatedness as a result of competitive exclusion in our simple homogeneous environment. We expected that this decline would be more pronounced at high relatedness, where the success of a strain is less affected by interactions with other clones and the fittest strain will be able to out-compete other strains. Further, we expected to see higher genetic diversity in the low relatedness populations, where the added complexity of the social environment provides a way for other clones to succeed. Specifically, we expected that social cheaters would be better able to take advantage of other cooperators at low relatedness. This expectation assumes that there is some trade-off between the two ways of achieving higher fitness.

## Results

### The social environment affects clone survivorship with high repeatability

Relatedness within fruiting bodies was significantly higher under high relatedness conditions (0.82 ± 0.08, mean and 95% CI) as compared to low relatedness conditions (0.31 ± 0.10) with no significant block effect, as determined by a fixed factor ANOVA (relatedness: *F*_*1,133 *_= 70.54, *p *< 0.0001, block: *F*_*2,133*_= 1.34, *p *= 0.265).

**Figure 2:**
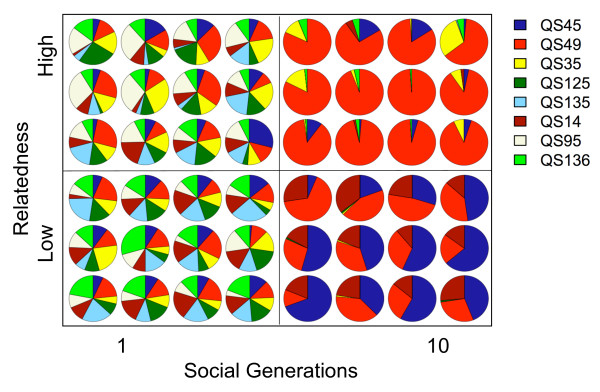
**Strain composition changed over time**. The frequencies of each strain in twelve populations after one and after ten social generations at high and low relatedness. The eight strains are represented by different colors (QS45 navy blue, QS49 red, QS35 yellow, QS125 dark green, QS135 light blue, QS14 maroon, QS95 white, QS136 light green). Each pie diagram represents a population.

After one social generation, all strains were present in all twelve replicate populations, both at high and at low relatedness (Figure 2). After ten social generations, however, a majority of the strains had decreased greatly in frequency and some had been completely eliminated. At high relatedness, one strain (QS49 red) overwhelmingly predominated in all twelve populations. Two strains (QS95 white and QS125 dark green) were no longer present in any population. One strain (QS135 light blue) was present in only one population and another strain (QS14 maroon) in three populations. At low relatedness, three strains (QS49 red, QS45 navy blue and QS14 maroon) predominated in every one of the twelve populations. On average, the frequencies of the three persisting strains increased significantly above their starting frequency of 1/8 (t-test on the difference between the final frequency and the initial frequency (QS45 and QS49 both *p *< 0.0001 and QS14 *p *= 0.001, d.f. = 11 for all t-tests; the increases all remain significant after applying sequential Bonferroni correction [21]). Two strains (QS35 yellow and QS125 dark green) were both present in three populations at very low frequencies. Three strains (QS95 white, QS135 light blue and QS136 light green) could not be detected in any of the twelve low relatedness populations.

**Figure 3:**
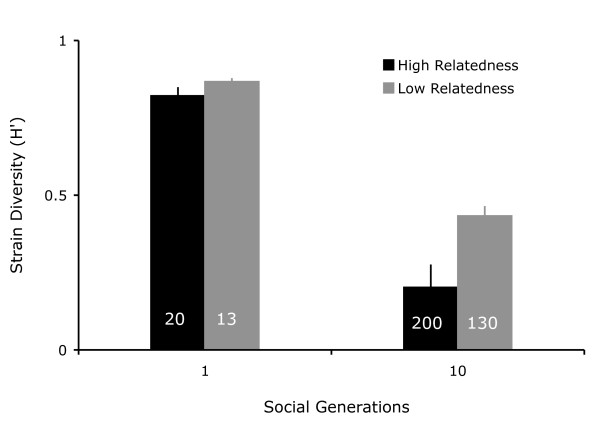
**Genetic diversity decreased over time**. Genetic diversity (H') decreased significantly over the course of the selection experiment. After one social generation, genetic diversity was high both at high and low relatedness with a small but significant difference between high and low relatedness populations. Over the course of the selection experiment genetic diversity decreased significantly more at high relatedness. Genetic diversity was measured as Shannon's Index of Diversity (H') and scaled to the maximum H' possible for eight strains (where p_i _= 1/8). Bars represent the mean of twelve populations with 95% CI. The numbers in the bars indicate the approximate numbers of vegetative generations (doublings) for each treatment after one and after 10 social generations.

Relatedness significantly affected strain composition and genetic diversity in the populations (Figure 3: *F*_*1,44 *_= 45.15, *p *< 0.0001), with a significant decrease between social generations 1 and 10 (*F*_*1,44 *_= 597.68, *p *< 0.0001) as determined by a full factor ANCOVA with relatedness and generation as fixed factors. More importantly, the decrease in genetic diversity was significantly stronger at high than at low relatedness, as indicated by a significant interaction term between relatedness and social generation (*F*_*1,44 *_= 10.58, *p *= 0.002). A test for normality of the residuals indicated that residuals were not normally distributed (Shapiro-Wilk test: *W *= 0.949, *p *= 0.038), which seemed to be driven mostly by one of the replicated populations (Population HR7). To test whether this population could affect the significance of the result, we repeated the analysis with HR7 excluded. This resulted in normally distributed residuals (Shapiro-Wilk test: *W *= 0.961, *p *= 0.117), without qualitatively changing the results of the ANCOVA.

### Strains that increased in frequency produced more spores and formed fruiting bodies more quickly than the other strains when grown in isolation

**Figure 4:**
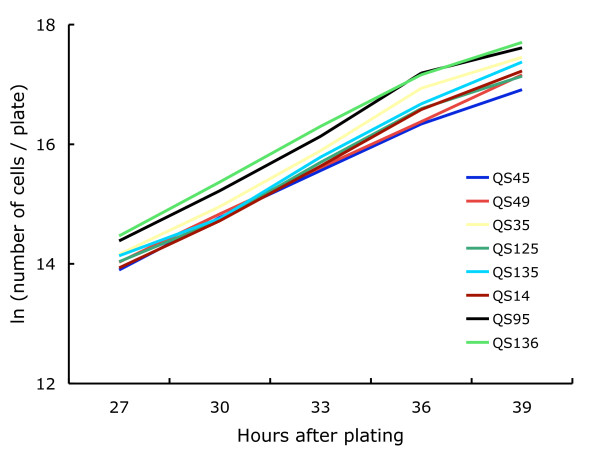
**Vegetative growth rates did not differ**. The eight strains did not differ in their vegetative growth. We counted the number of cells/plate every three hours between 27 and 39 hours after plating spores. To linearize the exponential growth data, we calculated the ln(number of cells per plate).

To assess the effects of life history traits, we grew each of the eight strains in the absence of the other strains and measured vegetative doubling rates, germination rates, spore production, and duration of the unicellular stage. Vegetative doubling rates during exponential growth did not differ significantly among the eight strains as indicated by a non-significant interaction term of strain and time (*F*_*7,72 *_= 1.092, *p *= 0.3774) in a full factor ANCOVA with strain, time and block as fixed factors (Figure 4). Nor did any of the clones show any change in doubling rate with increasing density (quadratic terms of the 8 regressions on time were all non-significant, p > 0.7).

Germination rates varied significantly among the eight strains (strain: *F*_*7,21 *_= 2.71, *p *= 0.036, block: *F*_*3,21 *_= 32.9, *p *< 0.0001). However, the three winning strains did not germinate significantly differently from the five losing strains (contrast analysis on least square means: *F*_*1,21 *_= 0.63, *p *= 0.435), which suggests that the differences in germination rate cannot explain the persistence of the three winning clones.

**Figure 5:**
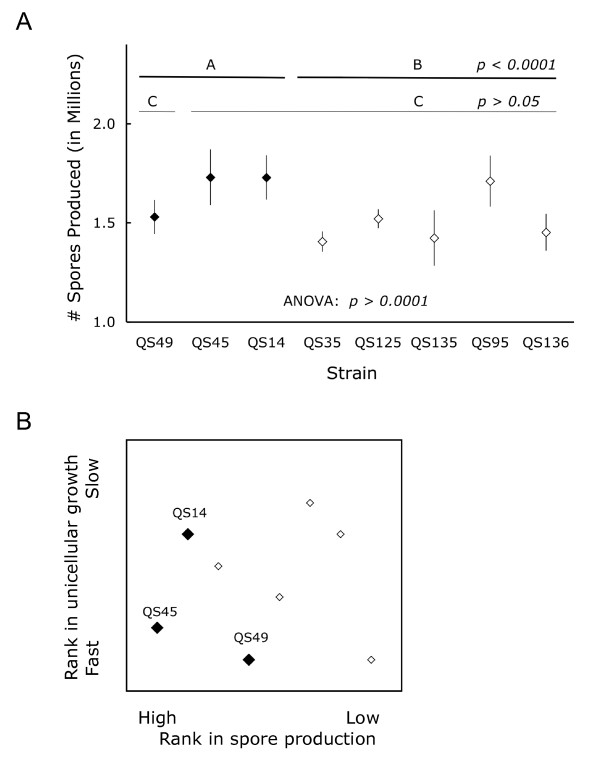
**Strains that persisted produced more spores and had a shorter unicellular stage**. Differences in total spore production as a measure of fitness among the eight strains. Points are mean of three measurements with 95% CI. The bars on top indicate the contrast analysis performed and their significance levels (A). Ranking of the strains based on spore production and progress during fruiting body formation relative to each other. A low rank indicates high spore production and fast fruiting body formation (B). Strains persisting at low relatedness (large, filled diamonds) tended to produce more spores and have short unicellular stage.

The eight strains differed significantly in spore production (genotype: *F*_*7,14 *_= 41.02, *p *< 0.0001, block: *F*_*2,14 *_= 3.15, *p *= 0.074; Figure 5a), with the three strains that increased in frequency over the course of the selection experiment producing significantly more spores from a constant starting number of spores than did the strains that decreased (contrast analysis on least square means: *F*_*1,14 *_= 101.65, *p *< 0.0001). However, the strain predominating in the high relatedness populations, QS49 (red), did not produce significantly more spores than the rest of the strains (contrast analysis on least square means of QS49 versus all other strains: *F*_1,14 _= 2.53, *p *= 0.13). This suggests that spore production affected the persistence of the three strains at low relatedness, but is not sufficient to explain the observed differences between high and low relatedness.

The eight strains differed considerably in their speed of fruiting body formation. When we plated equal numbers of spores in association with their bacterial food source on plates and scored the plates after three days of incubation, three strains (QS45 navy blue, QS49 red, and QS35 yellow) had developed farthest, to the stage called Mexican hat (Figure 1). Four strains (QS125 dark green, QS135 light blue, QS14 maroon, and QS95 white) were in the tight aggregate stage, and one strain (QS136 light green) was still in the loose aggregate stage. We used the speed of fruiting body formation as a proxy to estimate the length of the unicellular stage. Given that the strains did not differ in doubling rates, a shorter unicellular stage most likely arose from different threshold densities for aggregation. The three persisting strains all either produced more spores and/or formed fruiting bodies sooner than the other strains (Figure 5b). This suggests that a combination of high spore production and short length of the unicellular stage affected the strains' ability to persist over ten social generations and their ability to out-compete other strains. Differences in these traits however are not sufficient to explain the observed differences between high and low relatedness.

### Cheating in the social stage did not explain which strains persisted

**Figure 6:**
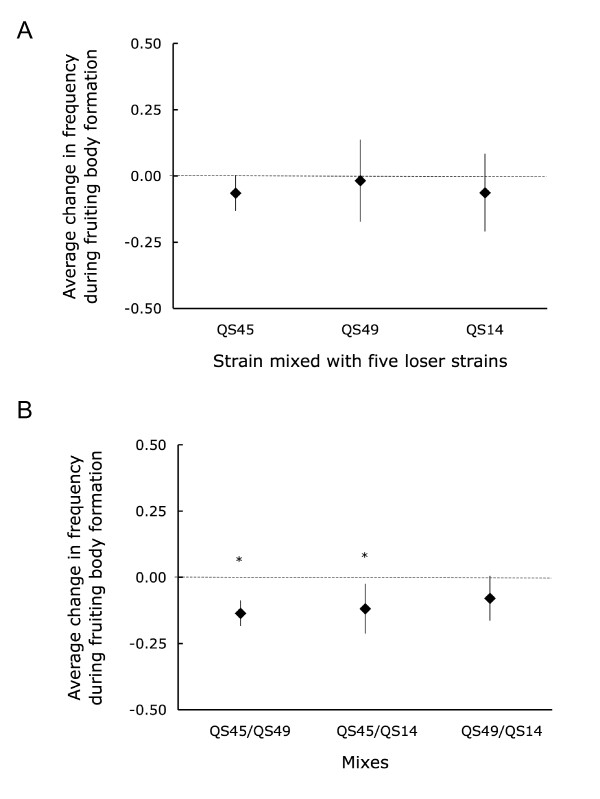
**The three strains that persisted at low relatedness did not cheat the other five strains but cheated each other**. Cheating assays of the persisting strains against the population of the five out-competed strains. The points represent the mean of three assays with 95% CI. All persisting strains tended decrease in frequency, though not significantly (A). Cheating assays among the three persisting strains. Each point represents the mean of six assays with 95% CI and is expressed as the change in frequency of the strain listed in bold (B).

We tested whether cheating, the overrepresentation of one clone in the spores relative to its initial frequency in the cells could account for the increase in frequency of the three strains at low relatedness by mixing starved cells of each of these three strains (QS45 navy blue, QS49 red, or QS14 maroon) with a mixture of starved cells of the five strains that decreased at low relatedness over the course of the selection experiment. All three of these strains showed an insignificant trend to decrease in frequency over the course of fruiting body formation (Figure 6a). Since we tested the directional hypothesis that the three strains would increase, we tested whether the change was significantly greater than zero. This effect was not significant for any of the strains individually (QS45 navy blue: *t*_*2 *_= -4.11, *p *= 0.97; QS49 red: *t*_*2 *_= -0.49, *p *= 0.67; QS14 maroon: *t*_*2 *_= -1.86, *p *= 0.90) or combined (*t*_*8 *_= -2.88, *p *= 0.99). The small sample sizes could potentially prevent detection of significance, but since all results went in the opposite direction from that expected for cheating, we think it is unlikely that lack of power was a problem. This suggests that cheating did not lead to the observed increase in frequency of the three strains at low relatedness over the course of the selection experiment.

Higher relatedness is expected to select for altruism over a greater range of benefits and costs [1]. As a result, we expected cheaters to be more likely to persist at low relatedness where they can gain an advantage when interacting with cooperators. To test whether some of the three strains persisting at low relatedness were able to cheat each other, we performed pair-wise cheating assays by mixing starved cells of two strains in equal proportions and counting the proportion of spores produced by each genotype (Figure 6b). We observed cheating among the three low relatedness strains, but found no evidence that the two clones that persisted only at low relatedness (QS45 navy blue, QS14 maroon) did so by cheating the one that was present at both high and low relatedness (QS49 red). QS45 (navy blue) was cheated by both QS49 (red: *t*_*5 *_= 7.28, *p *< 0.001) and QS14 (maroon: *t*_*5 *_= 3.26, *p *= 0.023). QS49 decreased in frequency when mixed with QS14, but not quite significantly so (*t*_*5 *_= 2.4, *p *= 0.062). We did not observe a significant change in frequency in the control mixes (labeled versus unlabeled), nor did we observe a significant block effect in either experimental or control mix.

## Discussion

Higher relatedness promotes the evolution of altruism all else being equal [1]. Spatial structure can limit interactions to neighbors and hence increase the likelihood that interactions are taking place among genetically similar individuals. To test the effect of relatedness on the evolution of altruism, we evolved genetically diverse populations for ten social generations at low and high density, which resulted in high and low relatedness, respectively. Over the course of the selection experiment, we observed a highly repeatable pattern: the same three strains increased in frequency at low relatedness and one of these three strains predominated in all populations at high relatedness.

The remarkably high degree of repeatability among the twelve replicated, independently evolved populations indicates that drift is not playing much of a role, even in the high-relatedness populations that started each round with only 500 spores. It is highly unlikely that the same strain would dominate in all twelve independently evolved populations solely due to chance (p < 0.0001: Binomial test for the dominance of one clone (p = 1/8) in twelve populations (k = 12) out of twelve populations (n = 12)).

Although low relatedness populations showed higher diversity, as we expected, social advantages during the fruiting body stage did not seem to have a strong effect. The three persisting strains did not cheat the other five strains as a group when mixed head to head. We expected that cheaters would be able to persist at low relatedness where they could exploit other strains and increase in frequency during the social stage. Not all of the three clones that persisted at low relatedness were equally successful in social competition with each other. However, one of the strains cheating during fruiting body formation was the most successful clone at high relatedness, indicating that it did not face a trade-off between the ability to cheat other strains and the ability to develop by itself.

Differences in spore production and length of the unicellular stage best explained which three strains persisted. Germination rate differed only slightly among the strains and could not account for the persistence of the three strains. Vegetative doubling rates did not differ measurably among the eight strains.

The observed difference in genetic diversity at high and low relatedness could be the result of factors other than relatedness. To achieve conditions of high and low relatedness, we plated the populations at low and high density, respectively. While we cannot completely rule out effects due to density-dependence, we found no evidence that density-dependent effects account for the different outcomes of our selection experiment. First, the eight clones do not differ measurably in their vegetative growth rates. Thus, no clone gains a significant advantage by having more cell generations in one of the treatments. The same data show that over a broad range of densities, each of our clones grows at a constant rate. We did not test the earliest stages, but at that time cells in both high-density and low-density treatments actually experience the same density, because they are all still in plaques started from single cells. Thus clonal vegetative fitnesses do not change with density, and different densities do not explain the differences between treatments.

**Figure 7:**
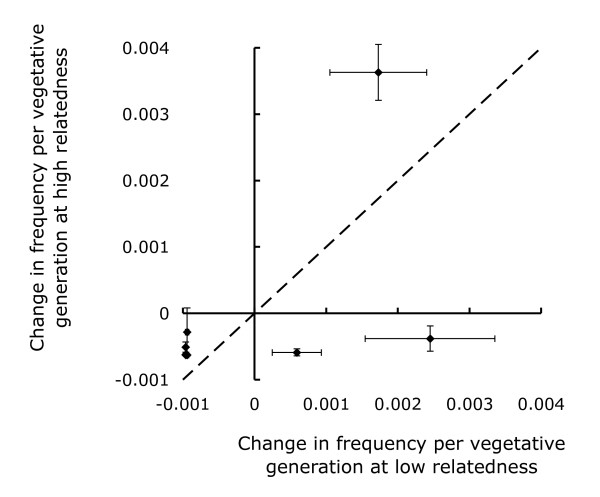
**Average change in frequency per vegetative generation are different at high and low relatedness**. The average change in frequency for each strain differed at high and low relatedness for the strains that persisted. Points represent the change in frequency (final - initial frequency (= 1/8)) divided by the number of vegetative generations (130 for low relatedness and 200 for high relatedness) of each strain averaged over twelve populations at high and at low relatedness with the 95% confidence intervals.

The difference in population size at transfer also resulted in more vegetative doublings during the unicellular stage in the high relatedness treatments. The average number of doublings per social generations was 13.4 at low relatedness and 19.7 at high relatedness, which is consistent with the 100-fold increase necessary to make up the difference in plating densities. Therefore, one could hypothesize that the higher genetic diversity at low relatedness is simply a transient state on a path that, if carried on farther, would yield the identical outcome as the high relatedness populations. Several lines of evidence suggest that this is unlikely to be the case. We did not observe any differences in doubling rates during unicellular growth. Therefore, it is unlikely that the additional doublings at high relatedness could account for the different strain compositions and diversities at high and low relatedness. In addition, over the course of the low-relatedness experiment QS45 (navy blue) and QS14 (maroon) increased from the starting frequency of 1/8, which would not be expected if they were on their way to being out-competed by the dominant one. More tellingly, the five losing clones were virtually eliminated from the low relatedness populations (mean summed population frequency 0.0054). If these populations were intermediates on the way to the dominance of QS49 (red) seen in the high relatedness populations, we would expect to see even fewer of these loser clones at high relatedness, but in fact we see many more (mean summed population frequency 0.093). This difference is significant (*p *= 0.002, d.f. = 13.15, *t *= 3.86, Welch t-test after arcsine transformation). Lastly, we calculated the average change in frequency of each strain per vegetative generation by dividing the difference between final frequency and initial frequency (= 1/8) by the number of vegetative generations (130 for low relatedness and 200 for high relatedness, respectively). If the difference in vegetative doublings accounted for the different outcomes at high and low relatedness, we would expect a correlation between the change in frequency at high and low relatedness after correcting for vegetative generations, which is clearly not the case, except for the one clone that did well under both conditions (Figure 7). Even so, we cannot definitively exclude a possible effect due to the different plating densities and the resulting differences in vegetative generations at high and low relatedness.

Gilbert et al. [4,5] assessed relatedness in natural populations and observed high levels of relatedness. High relatedness among interacting individuals can be achieved in two ways, by kin discrimination or by limited dispersal, and it is worth considering what role these mechanisms may have played in our experiment. *D. discoideum *clones are sometimes able to discriminate against other clones. In experimental mixes with wild clones, cells from genetically distinct strains sorted more with increasing genetic distance, while genetically similar strains formed chimeric fruiting bodies [22]. Benabentos et al. [23] recently identified high levels of polymorphism among wild isolates in members of the *lag *(recently renamed *tgr*) gene family which encodes trans-membrane proteins. A strong correlation between sequence divergence in these *lag *genes and sorting during fruiting body formation supports the hypothesis that these genes are important in kin discrimination. A preference for forming fruiting bodies with kin was also observed in other Dictyostelid species. Different genotypes of *Dictyostelium purpureum *preferentially aggregated and formed fruiting bodies with their clone mates [24,25]. Despite some ability to discriminate, our low-relatedness treatment did generate low relatedness among spores within fruiting bodies, and generated complete mixing in 1:1 mixes during the cheating assays (see Material and Methods) indicating that the eight strains used readily form chimeric fruiting bodies. This suggests that kin discrimination did not play an important role in our experiment as genetically different strains readily mixed and formed chimeric fruiting bodies when given the opportunity.

Limited dispersal could also promote interactions among genetically similar individuals [1]. Over the course of the life cycle, *D. discoideum *has different means of dispersal. During the unicellular stage, single cells can move short distances to explore new food sources. This kind of movement is expected to be the same in the high and low relatedness treatments. However in the high-density, low relatedness treatment single cells are more likely to encounter and compete with genetically different cells and it is this difference that we exploited to get high versus low relatedness treatments.

During the multicellular stage, *D. discoideum *forms slugs that move towards light and away from ammonia [26,27]. These slugs can move longer distances than single cells. Chimeric slugs have been observed to move less far than clonal slugs of the same size [28] and this effect would differ between our treatments. During the experiment, we minimized the effect of slug dispersal by keeping the plates in the dark without a light source. Long-range dispersal of *D. discoideum *can occur when spores are picked up by animals [4,5] or are washed away by water. In our experiment we collected all the spores after one social generation and thoroughly mixed the spores before plating them onto a fresh bacterial lawn for the next social generation. Therefore uncontrolled dispersal of slugs and spores did not have any effect on the structure of the next generation or on the different outcomes at high and low relatedness.

Cooperating individuals are very susceptible to exploitation by cheaters. Therefore it is difficult to explain the maintenance of cooperation in the light of cheating. One possibility is that cheaters have reduced fitness when they are unable to exploit cooperators and have to interact among themselves. Cheating assays among the persisting strains at low relatedness confirmed the presence of cheating. One of these cheaters (QS14 maroon) was unable to persist at high relatedness, suggesting such a cost to cheating. However, cheating did not seem to be costly for the other cheater (QS49 red), which dominated all high-relatedness populations. Cheaters unable to persist at high relatedness have previously been observed in *D. discoideum*. A known cheater mutant (*fbxA*^- ^[4] or *chtA*^- ^; [29]) was mixed with its wild type ancestor and while able to invade at low relatedness was unable to do so at high relatedness [4]. This inability to invade when the mutant was at high levels of relatedness is a result of the high fitness cost of the cheater mutation. When grown in isolation, this cheater mutant was not able to form fruiting bodies [4]. However, not all cheater clones experienced the same costs to cheating in our experiment. This is consistent with previous results, where we identified a large set of cheater mutants in *D. discoideum *[30]. Some of these cheater clones had a cost in terms of spore production when on their own, while others performed the same as the wild type, and still others produced even more spores than the wild type. All of these cheaters were able to form fruiting bodies with normal appearances on their own.

Our results suggest that trade-offs between cheating and the ability to form fruiting bodies alone vary among strains and that a combination of other life-history traits can partly explain the outcome of the selection experiment. Even so, interactions other than the ones we tested may contribute to shaping these populations. For example, we only tested pair-wise interactions during the multicellular stage. It is possible that the cheating dynamics change when all three strains can interact. Alternatively, our results may suggest that some interesting social interactions occur during the unicellular stage. Since the vegetative stage is quite long, small effects early in the vegetative stage could result in a large effect over the course of one social generation [20].

## Conclusions

For *D. discoideum*, spatial environment and the resulting differences in relatedness among interactors imposed strong selection pressures, as indicated by the highly repeatable differences in genetic diversity and strain composition between high and low relatedness. As expected, cheaters persisted better at low relatedness than at high relatedness. Interestingly, the strain that predominated in all populations at high relatedness was one of the cheaters. This indicates variable trade-offs between the ability to cheat other strains and the ability to form fruiting bodies alone. The persistence of the three strains was best explained by high spore production and a short unicellular stage, both traits that are strongly affected during vegetative growth. However, none of the traits examined could account for the difference between high and low relatedness, which suggests that other possible interactions occur during the vegetative stage. Our results suggest that the costs and benefits of the social interactions vary among strains and that social interactions in this social amoeba are complex and extend into the vegetative stage.

## Methods

### Strains

We used eight wild isolates of *Dictyostelium discoideum*, (QS45, QS49, QS35, QS125, QS135, QS14, QS95, QS136). These strains were isolated from soil samples collected along a 25 m transect near Mountain Lake Biological Station in Virginia (N 37° 21', W 80° 31') [31] and could be uniquely identified by a combination of two microsatellite loci using primers Dict13.CAT and Dict23.AAC [31].

### Manipulating Relatedness

To promote interactions at two different degrees of relatedness, we plated spores at low relatedness (LR: 50,000 spores/plate), or at high relatedness (HR: 500 spores/plate) on SM plates (10 g glucose, 10 g Bacto Peptone, 1 g yeast extract, 1 g MgSO_4_, 1.9 g KH_2_PO_4_, 0.6 g K_2_HPO_4_, 20 g agar per 1 liter H_2_O) with *Klebsiella aerogenes *as the bacterial food source. We verified that this manipulation actually impacted relatedness among spores within fruiting bodies as follows: To assess the average degree of relatedness within fruiting bodies for each treatment, we mixed spores of all eight clones at equal frequency and plated the mixed populations at the experimental high and low relatedness conditions (500 and 50,000 spores/plate, respectively). After fruiting body formation at the end of the multicellular stage, we collected single fruiting bodies, plated the spores from each fruiting body at very low density and genotyped single spores by isolating DNA from single clearings. We calculated the overall relatedness within fruiting bodies [32] for each experimental treatment using the software Relatedness 5.0.8 http://www.gsoftnet.us/GSoft.html. We estimated the probability above random chance that spores from the same fruiting body were clone-mates. We considered clone-mates to be related by 1 and non-clone-mates to be unrelated (0). To get a good estimate of relatedness within fruiting bodies, we chose to increase the number of fruiting bodies rather than the number of individual spores sampled per fruiting body and sample four spores per fruiting body for up to 24 fruiting bodies per block. We conducted the experiment in three temporally independent blocks and assessed a total of 66 and 71 fruiting bodies at low and high relatedness, respectively.

### Selection Experiment

We mixed spores of the eight clones in equal proportions and started two sets of twelve replicate populations by plating 500 spores/plate for high relatedness and 50,000 spores/plate for low relatedness on SM plates in association with a bacterial food source. During the selection experiment, we allowed the populations to grow and form fruiting bodies. Four days (LR) or five days (HR) after plating, we harvested the spores, and transferred the appropriate number of spores to a new plate with bacteria to initiate the next social generation. The time interval for harvesting spores differed between low and high relatedness because social aggregation occurred more rapidly in the more dense low relatedness populations. On average, the high relatedness populations went through 20 binary fissions during the unicellular stage of the life cycle and the populations at low relatedness through 13. After harvesting the spores, we plated a sample of each population at the appropriate plating density for the next social generation. We supplemented the remaining spores with glycerol and froze a sample of each population at -80°C. We repeated this process for a total of ten social generations.

At every transfer, we counted the number of spores produced by each population. The number of doublings each population went through during one social generation was calculated as the log of the base 2 of the total number of spores retrieved/number of spores plated.

### Assessing genetic diversity within populations

To assess the frequency of each strain in a population, we plated the populations at very low density (approximately 50 spores per plate) and collected cells originating from a single spore. We placed the cells into 20 μl of lysis buffer [33] to make DNA available for PCR and genotyped the cells at two microsatellite loci using primers Dicty13.CAT and Dicty23.AAC [31]. We genotyped up to 96 spores for each population. Some samples either did not amplify clearly enough to allow strain identification, or contained more than one strain, and were excluded from subsequent analysis. In total, we sampled between 55 and 96 spores per population after one (LR: 65.92 ± 3.46 (mean number of clones genotyped in 12 populations with standard deviation) and HR: 61.66 ± 3.63) and after ten social generations (LR: 92.75 ± 1.82; HR: 90.16 ± 4.87).

To assess genetic diversity within populations over the course of the experiment, we calculated Shannon's Index of Diversity as

where *p*_*i *_is the frequency of the *i*th genotype in the population. We scaled every estimate of H' to the maximum Index of Diversity possible for eight clones (H' _max, 8 clones _= 0.9031). H' is maximized when all genotypes are at equal frequency and decreases with decreasing number of genotypes or uneven frequencies. To assess the change in genetic diversity after 1 and 10 social generations and between high and low relatedness we performed a full factor ANCOVA using Shannon's Index of Diversity with social generations and relatedness as fixed factors. We arcsine transformed the data, which achieved normality of the data set and was appropriate for data ranging from 0 to 1. We used R [34], http://www.r-project.org for all statistical analyses unless otherwise noted.

### Vegetative doubling rate

To assess differences in vegetative doubling rates during exponential growth, we grew each strain separately by plating 50,000 spores per plate in association with a bacterial food source on replicated plates. After 27 hours of growth, we collected all the cells of a plate, diluted the cells in 10 ml of KK2 and counted the number of cells with a hemocytometer. We repeated this process for plates grown for 30, 33, 36 and 39 hours. We conducted the experiment in three temporally independent, complete blocks. To analyze the data, we log transformed the counts and performed a full factor ANCOVA with strain, time and block as fixed factors.

### Germination rate

To test for differences among germination rates, we plated each strain on six replica plates at a very low density of 50 spores per plate and counted the number of plaques formed after two days. We calculated the percent of spores germinated by dividing the number of plaques by number of spores plated on all six plates. We performed the experiment in four temporally independent blocks. To analyze the data, we arcsin-transformed the data and performed an ANOVA with strain and block as fixed factors.

### Total spore production in the absence of other clones

To determine the spore production of each strain, we plated 500,000 spores of each strain individually on 13 mm black filter squares (AABP 04700, Millipore, Bedford, MA) on nutrient-free agar (0.198 g KH_2_PO_4_, 0.036 g Na_2_HPO_4_, and 15 g agar per Liter H_2_O). We suspended and plated spores in KK2 buffer [22] with dead bacteria (OD_600 _= 6.0). We used dead bacteria as the food source to standardize and control the amount of food provided. Within four days, all strains had formed fruiting bodies. To assess the total number of spores produced, we collected the filters, placed them in 1 ml of KK2 buffer supplemented with 0.1% NP40-alternative (Calbiochem, La Jolla, CA), vortexed the tubes briefly to evenly disperse the spores before counting them. We conducted the experiment in three temporally independent blocks with two replicates per block. We performed a fixed factor ANOVA with genotype and block as factors on the means of the replicate plates per block with a subsequent contrast analysis on the Least Square Means (in JMP IN, Version 5.1.2) between different sets of strains.

### Aggregation and fruiting body formation during the multicellular stage

To test for differences in the length of the unicellular stage among the eight clones, we assessed how quickly the strains enter the multicellular stage. We plated spores of each strain individually in association with bacteria on SM plates and scored the plates after all strains had initiated the multicellular, social stage. We assumed that strains that were further along in fruiting body formation started the multicellular stage earlier and therefore had a shorter unicellular stage. Since the duration of the unicellular stage depends on the amount of resources available, we also scored the progress during fruiting body formation of the strains relative to each other, when we plated spores of each strain individually on filter pads with a standardized amount of dead bacteria. The progression during the multicellular stage of individual clones relative to the other clones was comparable when plated on SM plates in association with live bacteria to their relative progression on filter pads with dead bacteria.

### Cheating Assays

To test for cheating, we mixed cells of two competitors in equal frequencies and assessed the frequencies of the competitors before and after fruiting body formation. To test whether cheating could affect the success of certain strains, we mixed cells of a strain that increased in frequency with cells from a mixture of the strains that decreased in frequency over the course of the selection experiment. Before mixing the two competitors, we created a mixed population of the strains that decreased over the course of ten social generations by mixing spores of these strains in equal proportions and plating them in association with bacteria. We harvested the cells of the mixed population and cells of the competitor during exponential growth, labeled and set up the mixing experiments as described in Ostrowski et al. [22]. After 24 hours of incubation, we collected the spores into 5 ml of KK2 with detergent (1.36 g KH_2_PO_4_, 1.74 g K_2_HPO_4_, 5 ml of 10% NP-40 Alternative (Calbiochem, La Jolla, CA), 1.86 g EDTA in 500 ml, pH = 7.1) and assessed the frequency of labeled and unlabeled spores. To assure the health of the cells during this time intensive experimental set-up, we labeled the cells of the mixed population and mixed these labeled cells with unlabeled cells of each strain that increased over ten social generations. We conducted the experiment in three complete, temporally independent blocks, which allowed us to compete each strain against the same mixed population. To test for the effect of labeling, we added a control by mixing labeled and unlabeled cells of the mixed population of the decreasing strains in equal proportions and assessed the frequency of labeled spores. Cheating was assessed as a significant change in the frequency of each competitor during fruiting body formation. We determined the cell and spore frequencies of each strain at the beginning and end of the multicellular stage, respectively. The change in frequency was calculated as the difference between spore frequency minus cell frequency. For the analysis, we performed a one-tailed t-test for each of the three strains individually and combined to test whether the persisting strains cheated the other strains by out-representing themselves in the spores.

We performed a second set of cheating assays using only the strains that increased in frequency, following the protocol described in Ostrowski et al. [22] in three complete blocks with reciprocal, pair-wise mixes. Since we were interested in cheating, it was essential that the mixed strains actually formed chimeric fruiting bodies. To confirm this, we counted ten individual fruiting bodies for each experimental and clonal mix of the first block. To test for sorting, we calculated the relatedness, r, within fruiting bodies as

where *p*_*i *_is the frequency of a strain in the fruiting body and  is the frequency of the same strain in the total population. The frequency in the total population was calculated as the frequency of the strain across all ten fruiting bodies. Relatedness is scaled between 1 and -1, with 1 and -1 indicating complete sorting and high relatedness within fruiting body, and zero indicating completely random mixing. Overall, relatedness within fruiting bodies was very low (0.0078 ± 0.018, mean and 95% CI), indicating that the strains mixed and formed chimeric fruiting bodies. To test whether the mixing in the experimental mixes differed significantly from the control mixes (labeled and unlabeled cells of the same strain), we performed an ANOVA with treatment as a fixed factor and did not observe evidence for sorting of clones into different fruiting bodies. We could not detect a difference in relatedness within fruiting bodies between the control mixes and the experimental mixes (*F*_*1,116 *_= 0.0826, *p *= 0.77). This indicated that the strains readily formed chimeric fruiting bodies. Therefore, we assessed the frequencies of the two strains in the total population to get an estimate of cheating.

We assessed cheating as described above. Since the cells used for reciprocal labeling were collected independently, we pooled the data from reciprocal mixes by expressing the change in frequency in terms of the same clone. To test for a significant change in frequency, we performed a one-sample t-test (two sided) on the change in frequency from cells to spores. To test for significant block effects, we performed an ANOVA with strain and block as factors. Since we used the difference and not the actual frequency data, we did not need to transform the data.

## Abbreviations

HR: high relatedness; LR: low relatedness.

## Authors' contributions

GS, JES and DCQ conceived and designed the study. DAB designed and performed the experiment testing the total spore production. GS performed all other experiments and analyzed the data. GS, DAB, JES, and DCQ wrote the manuscript. All authors read and approved the final manuscript.
